# Evaluating work activity and societal burden in patients with grade 2 IDH-mutant glioma

**DOI:** 10.1093/nop/npaf092

**Published:** 2025-09-01

**Authors:** Slávka Lukacova, Aida Muhic, Oskar Ström, Morten Johnsen, Céline Aubin, Marc Massetti, Jane Skjøth-Rasmussen

**Affiliations:** Department of Clinical Medicine, Aarhus University, Aarhus, Denmark; Department of Oncology, Aarhus University Hospital, Aarhus, Denmark; Department of Oncology, Copenhagen University Hospital, Rigshospitalet, Copenhagen, Denmark; Department of Medicine, Huddinge, Karolinska Institutet, Stockholm, Sweden; Quantify Research, Stockholm, Sweden; Quantify Research, Stockholm, Sweden; Servier International, Suresnes, France; Servier International, Suresnes, France; Department of Clinical Medicine, Copenhagen University, Copenhagen, Denmark; Department of Neurosurgery, Neuroscience Center, Copenhagen University Hospital – Rigshospitalet, Copenhagen, Denmark

**Keywords:** glioma, IDH mutation, work activity, costs, survival

## Abstract

**Background:**

IDH-mutant (mIDH) gliomas affect relatively young and otherwise healthy patients with high workforce participation. Management typically involves surgery, radiotherapy, and/or chemotherapy (RT/CT), although immediate post-surgery treatment may be deferred in favor of active observation (AO) to preserve functioning. This study aimed to evaluate how disease progression and treatments impact work activity, medical and societal costs, and overall survival (OS) in grade 2 mIDH glioma patients initially managed with AO.

**Methods:**

This retrospective longitudinal study utilized Danish administrative registries. Patients with mIDH gliomas initially managed with AO between 2010 and 2022 were included. Employment rates, sick leaves, treatment patterns, medical resources and costs, and OS were examined from diagnosis to the end of follow-up or death.

**Results:**

Data from 237 patients were analyzed; 72.9% were alive at 10 years. After surgical recovery, ability to work was most impaired during RT/CT, necessitating nearly full-time sick leave. Work activity continuously decreased after each RT/CT treatment. Employment rates were similarly affected, from under 20% unemployment at baseline and during AO, up to 41% and 59% after first and second RT/CT. Similar trends were observed for medical resources and costs, and productivity losses.

**Conclusions:**

Patients with grade 2 mIDH glioma remain active at work after initial surgery when managed with AO. Work impairment increases over successive RT/CT courses, highlighting the need to preserve quality of life by integrating work ability into clinical practice and by developing new treatment strategies to delay aggressive therapies and avoid substantial medical and productivity costs.

**Key Points**

IDH-mutant gliomas significantly impact work activity, throughout progression and treatments.
Following radiotherapy and/or chemotherapy, many patients are unable to return to full-time employment, while some cannot resume work at all, leading to substantial productivity losses.

**Importance of the Study**

Few studies report on patients’ ability to work in glioma patients, especially beyond the post-surgery setting. This study reports work activity, healthcare resource utilization, treatment patterns, and societal costs associated with grade 2 IDH-mutant (mIDH) glioma patients managed with active observation (AO) after initial surgery, leveraging comprehensive nationwide patient-level Danish administrative registers. Our results highlight the association of work activity with disease progression and treatments throughout the patient pathway. These findings are associated with substantial financial burdens on healthcare systems during post-surgery and treatment periods. Additionally, the study includes overall survival for a nationwide cohort of grade 2 mIDH patients initially managed with AO. These insights underscore the importance of integrating work ability considerations into clinical practice and developing novel treatment strategies allowing to delay unequivocal need for initiation of radiotherapy, and/or chemotherapy, thereby preserving patients’ quality of life and reducing economic costs.

Gliomas account for 77% of over 250 000 new cases of primary malignant brain tumors diagnosed annually, with an age-adjusted incidence rate of around 5.8 per 100 000 in the United States.^1^ Registry studies from Norway and Denmark have reported comparable incidence rates of approximately 7.1 and 7.3 per 100 000 person-years, respectively.^2^

Recent advancements in molecular diagnostics have greatly improved the classification of gliomas, incorporating genetic markers into the World Health Organization (WHO) classification system.^3^ Consideration of key molecular markers, such as mutations in isocitrate dehydrogenase (IDH), 1p/19q codeletion, and TP53/ATRX status, has led to improved diagnosis, allowing for more precise treatment and prognostics.^4^

IDH-mutant (mIDH) diffuse gliomas are defined by mutations in the genes encoding the metabolic enzymes IDH 1 or IDH 2 and account for 7.3% of all brain tumors in the United States.^5^ They include astrocytomas (mIDH) and oligodendrogliomas (mIDH, 1p19q co-deleted) and are typically initially slow-growing tumors that eventually progress to a higher grade of malignancy.^6^

Incidence of mIDH gliomas increases with age and peaks between 30 and 34 years for astrocytomas and between 40 and 44 years for oligodendrogliomas, with incidence subsequently decreasing with advancing age. While comparable in patients with mIDH grade 2 or grade 3 tumors, the median overall survival (OS) significantly varies between histologies, ranging between 5^1,7^ and 8^8,9^ years in astrocytomas, versus 13^7–9^ and 17^1^ years in oligodendrogliomas.

Surgery represents the basis of disease management with the aim to achieve maximal safe resection, providing a definitive diagnosis, facilitating molecular analysis, reducing seizures, while preventing neurological deficits to maintain QoL, and improving survival outcomes.^10^

After initial surgery and integrated diagnosis, the choice of initiating an oncologic treatment is influenced by several factors (eg, tumor grade, type, size and location, postoperative tumor volume, age and performance status, neurological symptoms, seizure burden, and patient preferences), aiming to balance quality of life (QoL) with effective tumor control, and includes active observation (AO), radiotherapy, or combined radiotherapy and chemotherapy (RT/CT).

Combination of RT/CT (eg, Temozolomide or PCV) has shown significant survival benefits over RT alone,^8,9,11^ establishing it as standard of care in clinical guidelines,^12–14^ though no curative treatment exists.^15,16^ However, the optimal timing of RT/CT remains unclear. Results of meta-analysis involving 311 participants with diffuse glioma found that early postoperative radiotherapy was associated with an increase in time to progression compared to AO (and delayed radiotherapy upon disease progression), but did not significantly improve OS.^17,18^ Therefore, in patients with good prognostic factors (eg, grade 2, gross total resection, oligodendroglioma, and no neurological symptoms), postoperative RT/CT may be deferred in favor of AO to avoid radiation-induced neurocognitive decline and chemotherapy-associated side effects.^13,19–21^

The recently approved mIDH1/2 inhibitor Vorasidenib demonstrated significant benefits over placebo in terms of progression-free survival and time to next intervention. Vorasidenib has been included in clinical guidelines for managing patients with mIDH diffuse gliomas for whom treatment with RT/CT is not indicated.^22–25^ Living with mIDH glioma profoundly impacts those of working age, as disease progression and debilitating effects of treatments hinder patients’ ability to work.^26^ Although work-related challenges are often highlighted by patients^27–29^, existing research on work activity with glioma is limited, primarily focusing on the post-surgery period and treating return-to-work as an outcome of surgery rather than its association with disease progression and treatments, with rates ranging between 63% and 94%.^30–33^ Thus, the impact of mIDH glioma diagnosis and nonsurgical treatments on work activity and societal costs during the entire course of the disease is not well documented.

Tracking work activity is a valuable approach to better understand how the disease affects the patients’ daily functioning, as well as the societal costs associated with the disease.^34^ Danish administrative registers offer a unique opportunity for combining disease trajectory data from the administrative healthcare registries with workforce participation data from the DREAM register to assess work ability and sick leaves during multiple disease periods.^35–37^

This study aims to utilize Danish registries data to examine the impact of disease progression and treatments on patients with grade 2 mIDH glioma who are not in immediate need of RT and/or CT after initial surgery, focusing on work inactivity, healthcare resource utilization (HCRU), costs, and OS.

## Methods

### Study Design and Setting

The study period spanned from January 2008 to March 2024. This non-interventional, observational, retrospective longitudinal study utilized pseudonymized patient-level data from multiple Danish administrative registries to identify grade 2 mIDH glioma patients not in immediate need of RT/CT after initial surgery and followed their workforce participation prior to diagnosis and through their course of disease. The study used an incident approach, where newly diagnosed patients were followed throughout consecutive lines of therapy until death, emigration, or end of follow-up (EOF).

### Data Sources

Workforce participations were captured through the Danish Register for Evaluation of Marginalisation (DREAM), which contains nationwide person-level weekly information on social transfer payments^38^ and monthly employment data for all working individuals with tax payments in Denmark, including self-employed individuals.^39^

The Danish National Patient Registry (NPR), registry of medicinal product statistics (PDR), cancer register, pathology register, hospital medication registry, and population register are nationwide person-level registers and were used for patient identification and characterization and assessment of comorbidities, treatments, medication, HCRU, and healthcare costs. Patients were linked and followed across those multiple registries through their unique pseudonymized social security numbers. Data sources and codes used to identify and select patients and assess the study outcomes are presented in **Supplementary Table 1**.

### Study Population

The study population consisted of grade 2 glioma patients with a primary neurosurgical procedure providing histologically confirmed evidence of oligodendroglioma or diffuse astrocytoma with mIDH who were alive, had no other prior cancer diagnoses, and were not in immediate need of RT/CT and/or not receiving palliative care (PC) after their first neurosurgical procedure. Patients were included if they met the following criteria:

1. A recorded oligodendroglioma or diffuse astrocytoma diagnosis in the Danish Cancer Register between January 1, 2010, and December 31, 2022.
2. A recorded neurosurgical procedure or a pathological examination requisition date, ensuring that patients with diagnostic surgery were not overlooked, even if recorded under different or nonstandard diagnostic surgery codes.
3. A morphology (M) and/or function (F) code indicating mIDH, with or without 1p/19q codeletion in the Pathology Register, with a requisition date dating between 2 months before and 6 months after the incident glioma diagnosis date.

The following exclusion criteria were applied:

1. A cancer diagnosis recorded in the Cancer Register (ICD-10 codes C00–99, excluding non-melanoma skin cancer and gliomas) prior to the pathological examination requisition date, and/or
2. Any of the following within 0–6 months after the pathological examination requisition date:

- RT recorded in the Patient Register
- CT recorded in the Patient Register or the Hospital Medicine Register
- PC recorded in the Patient Register
- Death

Data sources and codes used to identify and select patients are presented in **Supplementary Table 1**.

### Time Periods

Individual index dates were established based on the date of the first request for the IDH mutation examination in the Pathology Register. Patients were followed over a period of 21 months before their index date until post-index censorship due to EOF, death, or emigration, whichever happened first.

A pre-index period of 12 months, comprising the 21 to 9 months preceding the index date, was established to characterize patients’ baseline work-activity outcomes, HCRU, and health status.

Post-index date follow-up was split into different periods that matched the disease and treatment pathway of patients with mIDH glioma not in immediate need of RT/CT after initial surgery. Records of employment, sick leave, HCRU, treatments, time to next period, and survival were allocated to the corresponding periods:

- Post-surgery: A 3-month period following index date (initial surgery) and any subsequent record of surgery (re-surgery).
- AO: Period following the post-surgery period until the first record of the patient receiving RT/CT or PC or EOF.
- RT/CT treatment: Initiated upon record of RT/CT or PC and ending after no recorded evidence of RT/CT or PC or at EOF. First (“1st RT/CT” period) and subsequent lines of RT/CT were distinguished, considering all subsequent RT/CT lines after the first one as “≥2nd RT/CT” periods.
- Monitoring after RT/CT: Periods following last RT/CT or PC administration until next record of patient receiving RT/CT or PC, or EOF, distinguishing post-first RT/CT and post-≥second RT/CT periods.

The 3 months following the occurrence of surgeries were not considered  initiation of a new period. Instead, these events were considered as a 3-month post-surgery period nested into the ongoing period, meaning that patients could experience multiple surgeries over the same period. This was done to ensure that the specificities of the surgery and post-surgery setting would not bias the outcomes of other periods.

Definitions and timeframes corresponding to the study periods are available in **Supplementary Table 2**.

### Study Outcomes

Baseline characteristics included age, sex, and histology at index date, employment rate, monthly sick leaves, and Charlson–Quan Comorbidity Index^40^ during the pre-index period.

Work activity outcomes in nonretired patients between 16 and 64 years of age represented the primary objective of the study. They were estimated in each study period using the DREAM register based on employment rates and sick leave. Employment rate was measured relative to full-time employment consisting of 160.33 working hours per month, determining full-time (≥95%), part-time (>0%–94.99%), and unemployed (0%) statuses. Students were identified in the DREAM register and treated as being full-time employed for the duration of their studies. Sick leaves were estimated as weeks with sick leave per month based on recorded sick leave payments from the Danish municipalities. Sick leave and employment status were reported as the mean and median number of sick leave weeks per month (4.3 weeks), number of patients who were on sick leave 100% of time over the period, the percentage of the period spent on sick leave, as well as the distribution of patients by employment status during each period.

Productivity losses were derived from work activity outcomes using the human capital approach.^41^ First, the number of weeks absent from work by patient and period was calculated and adjusted individually based on patients’ employment rate 21 months before the index date. Then, the productivity loss was calculated by multiplying the additional weeks of sick leave in each period compared to the pre-index period by the average gross full-time salary in Denmark in 2023 (6522 €/month).

For each period, treatment patterns were described through the estimation of the average numbers of surgeries and RT/CT courses and their characteristics, as well as time to next period and reason for period end (next period, PC, EOF, or death).

HCRU, including the mean number of inpatient and outpatient visits, inclusive of surgeries and RT/CT administrations, was estimated from the National Patient Register. Healthcare costs were assigned to each visit using Diagnosis-related group tariffs (DRG grouped NPR). Prescription medications and hospital-administered drugs were retrieved from the Registry of Medicinal Product Statistics and the National Hospital Medication Register, respectively.

HCRU was associated with the occurring period at the time of the record, and average HCRU per patient and per month were estimated within each period. Inpatient and outpatient visits, surgeries, RT/CT administrations, hospital-administered drugs, and prescription medications were associated with their corresponding costs to estimate the respective medical costs per period.

Time to next line of RT/CT was assessed using Kaplan–Meier (KM) analysis and was defined as the duration from the index date or RT/CT until the next line of RT/CT, with death as a competing risk and EOF and emigration as censoring events.

OS was defined as the time from the index date to all-cause mortality.

### Statistical Analysis

Continuous values were reported as mean, median, IQR, and standard deviation, and categorical values were described by frequency and proportion. OS and time to next period were assessed using a KM approach.

Healthcare costs were adjusted to 2023 values using the Healthcare Consumer Price Index from Statistics Denmark. Danish crowns (DKK) were converted to Euros using the 2023 average exchange rate (7.45 DKK = 1.00 €).

## Results

Data from 467 patients diagnosed with glioma, 180 (39%) with an oligodendroglioma, and 287 (61%) with a diffuse astrocytoma, were retrieved. Among these, 439 had an IDH mutation recorded in the Pathology Register. Excluding patients with prior cancer diagnoses, 413 cases remained.

Out of these patients, 237 did not require immediate RT/CT, or PC, or died within 6 months after the index date and constituted the study cohort of mIDH grade 2 gliomas: 106 (45%) with oligodendroglioma and 131 (55%) with diffuse astrocytoma.

### Baseline Characteristics

Mean age at index was 40 years. Two hundred and four (86.1%) patients were between 16 and 64 years old and were not retired. Of these, nearly half were full-time employed, and 19% were unemployed pre-index. The population was generally healthy, with 97% having no comorbidity based on the Charlson index. Median follow-up duration was 6.1 years. Over this period, 104 (51%) of patients initiated a first line of RT/CT and 45 (22%) a second line. Median follow-up duration for the AO, post-first, and post-second RT/CT period was 3.4, 1.2, and 0.6 years, respectively. The censoring event was EOF in 79% of cases and death in 20% (Table 1).

**Table 1.** Baseline Patient Characteristics (*N* = 237)

Characteristic	*N* (%)
Age	
Mean (SD), years	40.3 (16.3)
Median (Q1–Q3), years	38 (29–50)
Distribution, *n* (%)	
Age <16	12 (5.1%)
Age 16–39	111 (46.8%)
Age 40–64	93 (39.2%)
Age 65+	21 (8.9%)
Histology, *n* (%)	
Diffuse astrocytoma	131 (55%)
Oligodendroglioma	106 (45%)
Sex, *n* (%)	
Male	112 (47.3%)
Female	125 (52.7%)
Charlson–Quan Comorbidity Index, *n* (%)	
0	229 (96.6%)
1+	8 (3.4%)
Employment status, *n* (%)	
16–64 and nonretired	204 (86.1%)
Full-time employed: ≥95% mean employment rate Part-time employed: >0–95% mean employment rate	91 (44.6%) 74 (36.3%)
Unemployed: 0% mean employment rate	39 (19.1%)
Follow-up	
Mean duration (SD), years	6.4 (3.2)
Median duration (Q1–Q3), years	6.1 (3.8–8.9)
Censoring event, *n* (%)	
Death	46-49 (19.4%–20.6%)^SD^
Emigration	1–4 (0.4%–1.7%)^DD^
End of follow-up	187 (78.9%)

Abbreviations: DD, direct disclosure to ensure patient anonymity; SD, secondary disclosure.

### Work Activity and Productivity Losses

**Figure 1.**
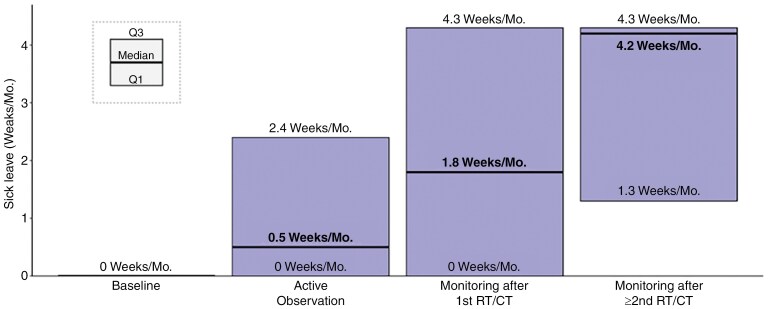
Median number of weeks of sick leaves per patient per month, during baseline, AO and monitoring after RT/CT periods

During the pre-index period, nonretired subjects (*N* = 204) required 0.3 weeks of sick leave per month on average [median (Q1, Q3) = 0 (0, 0)]. Post-index, the AO period was characterized by the lowest level of monthly sick leave weeks (median (Q1, Q3) = 0.5 (0, 2.4) week per month). Patients required increasing sick leaves as disease and treatment lines progressed, peaking during RT/CT treatment with 4.3 weeks of sick leaves per month in median (Q1, Q3) [first RT/CT: 4.3 (0,4.3); second RT/CT: 4.3 (0.7–4.3)], followed by the post-surgical period at 3.5 (1, 4.3) weeks per month. Furthermore, observation periods that followed the administration of RT/CT were associated with considerably higher sick leave frequencies than the AO period with medians of 1.8 (0, 4.3) and 4.2 (1.3, 4.3) monthly weeks of sick leave after first RT/CT and ≥second RT/CT lines, respectively (**Figure 1**).

**Figure 2.**
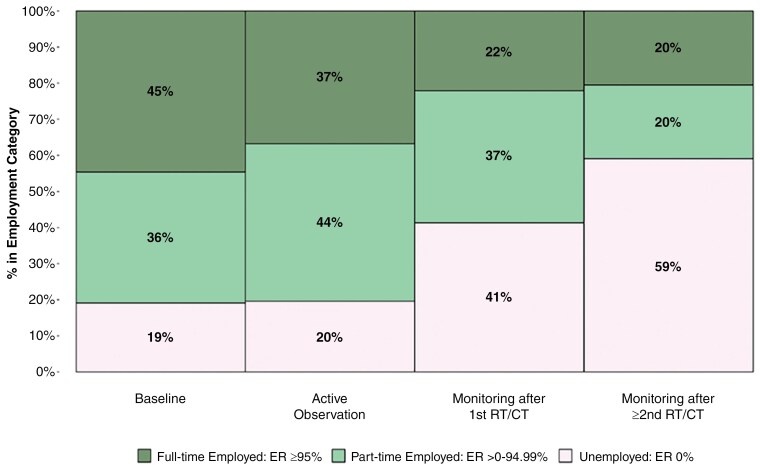
Mean employment rate during baseline, AO and monitoring periods after RT/CT. Key: Employment rate estimated as the number of working hours per month over a full-time of 160.33 working hours per month.

Employment rates were similarly affected by disease progression and oncological treatments from less than 20% patients unemployed pre-index and during AO (19.1% and 19.6%, respectively), doubling after first RT/CT (41%) and tripling after second RT/CT (59%), further emphasizing the challenges of work force participation that follows disease progression and treatment with RT/CT (**Figure 2**).

Together, these work force participation results determined increasing average monthly productivity losses of €1200 during AO, and €2250 up to €3300 during the monitoring periods following the first and second RT/CT.

### Healthcare Resource Use and Medical Costs

Consistent with the age and low comorbidity burden of the population, pre-index HCRU and corresponding medical costs were low (€277/patient/month on average). After the index date, the post-surgery, first, and second RT/CT treatment periods were associated with the highest monthly medical costs (respectively €9948, €10 125, and €14 634 per patient), although these periods are limited to a few months. Monthly medical costs also increased considerably from AO (€1076 per patient) compared with the monitoring periods following first (€3709) and ≥second (€4894) RT/CT, driven by more frequent and longer hospitalizations as well as increasing prescription medicine costs (Table 2).

**Table 2.** Healthcare resource utilization and costs per patient per month

	Pre-index	Post-surgery	AO	First RT/CT	Monitoring after 1st RT/CT	≥Second RT/CT	Monitoring after ≥Second RT/CT
*N*	237	237	237	115	115	49	48
Outpatient visits							
* n*	0.21	1.2	0.74	9.28	1.07	6.09	1.44
Average cost	€71	€546	€324	€8,477	€450	€11 263	€789
Inpatient visits							
* n*	0.02	0.45	0.06	0.18	0.12	0.17	0.32
Average cost	€67	€9097	€415	€1067	€2640	€2489	€3353
Average hospitalization days	0,06	2,78	0,24	2,07	0,84	1,02	1,54
Prescriptions							
Average cost	139 €	305 €	337 €	580 €	620 €	881 €	753 €
**Average total cost per month**	**277 €**	**9948 €**	**1076 €**	**10 125 €**	**3709 €**	**14 634 €**	**4894 €**

Abbreviations: AO, active observation; RT, radiation therapy; CT, chemotherapy.

### Treatment Patterns

Occurrence of re-surgeries was 48% between index and first RT/CT, <15% after first RT/CT and <20% after second. The proportion of patients receiving RT dropped from 74.6% at first treatment line to 15.6% at second treatment line. Whereas more than 80% of patients received CT across all treatment lines.

Median time to first RT/CT was 6.1 years from index date, and 5.2 years from initiation of first to second RT/CT. Time to next RT/CT was not available beyond second RT/CT.

### Overall Survival

**Figure 3.**
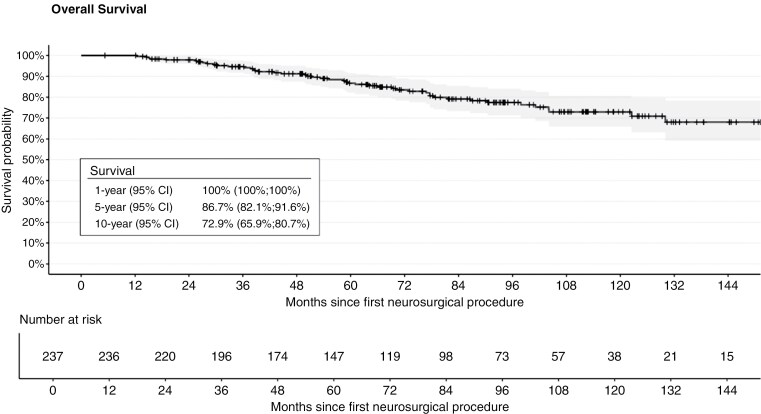
Overall survival. Patients with grade 2 mIDH glioma not in immediate need of RT and/or CT after initial surgery.

OS at 1, 5, and 10 years was 100%, 86.7%, and 72.9%, respectively (**Figure 3**).

**Figure 4.**
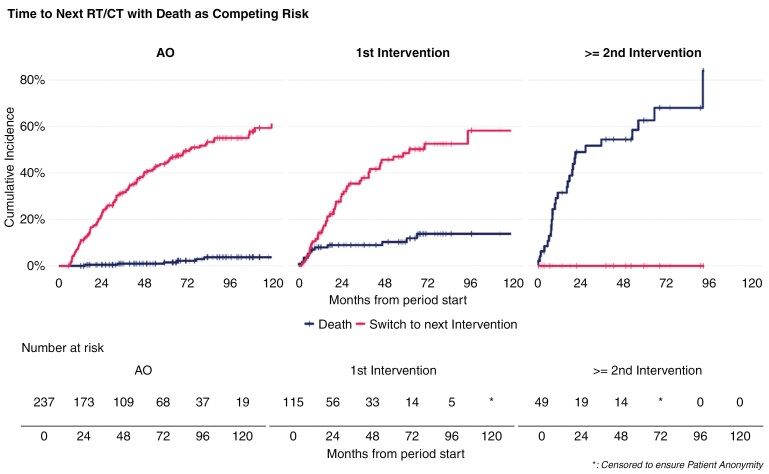
Time to next RT/CT with death as competing risk.

Results by period, with next RT/CT as a competing risk indicated low mortality until the second RT/CT, with overall cumulative incidence of death below 5% at 5 years in AO and 12% in first RT/CT, versus, respectively, 31.5% and 62.7% at 1 and 5 years from second RT/CT (**Figure 4**).

## Discussion

To our knowledge, this study is the first to examine the association between disease and treatment progression with workforce participation in grade 2 mIDH gliomas. We used pseudonymized patient-level Danish national registers to study workforce participation and the associated productivity losses during distinct time periods identified from the time of diagnosis in patients with grade 2 mIDH glioma not in immediate need of RT/CT. The study also investigated related topics, such as treatment patterns, healthcare resource use, healthcare costs, and OS in this population.

Compared with many other cancers, this population is young, relatively healthy, and has a high workforce participation, emphasizing the need to understand the impact of the disease and treatments on patients’ ability to work and integrate it into clinical practice. This is further accentuated by the a priori good prognosis and long expected OS in this population, as confirmed in our findings, where, respectively, 87% and 73% of patients were alive at 5 and 10 years. The study population was selected based on a criterion of 6-month survival, which limits direct comparability with existing literature. Nonetheless, the findings remain consistent with previously published data in mIDH patients initially managed with AO.^17,42,43^

Akin to available evidence in the peri-surgical setting,^30–33^ our results confirm that, after a recovery period characterized by highly impaired ability to work, patients managed with AO are able to maintain high workforce participation and pre-surgery levels of employment. While undergoing RT/CT, patients’ ability to work is severely impaired, with most patients not working during this period. Furthermore, our data highlights that ability to work remains altered during the monitoring phases that followed the completion of first RT/CT and particularly so after second RT/CT. This was characterized by increasing proportion of unemployment from 20% during AO (similar to baseline rates), up to 40% after first line of oncologic therapy and 60% after second RT/CT, while working patients required more sick leaves from 0.5 weeks every month in median during AO, to 1.8 weeks after first RT/CT, and 4.2 weeks after second RT/CT. Consequently, the societal economic burden associated with the disease increased during those periods, from a societal cost of €1200/month/patient during AO up to €3300 after the second RT/CT treatment. Incidentally, HCRU and related medical costs related to patients’ monitoring more than tripled after first RT/CT.

Ability to work was previously reported as a key concern for mIDH patients^27–29^ and was demonstrated as significantly associated with QoL.^44–50^ Indeed, as physical and mental well-being directly influence an individual’s capacity to perform and sustain employment, our findings collectively highlight that from the onset of RT/CT, patients’ ability to work and functioning has deteriorated irreversibly, leading to increased HCRU and a significant negative impact on society. Thus, delaying disease progression and the need for RT/CT is crucial to preserving the QoL for patients with mIDH gliomas.

Our study presents several limitations. Firstly, the follow-up duration and the censoring of patients before the first or second line of RT/CT may affect the generalizability of our findings. As attrition increases over time due to the reduction in available follow-up for patients diagnosed more recently, more advanced treatment and observation periods include fewer patients diagnosed in recent years, thus censoring the duration of the ongoing period at the end of their follow-up. This also implies a selection bias toward patients with faster progressing disease in the later periods, as those with more favorable characteristics (eg, oligodendrogliomas, no/low residual disease, favorable molecular features) were less likely to reach the later stages before the end of follow-up. Nevertheless, respectively, 37% and 31% of patients who reached the first and second line of RT/CT had oligodendrogliomas, compared to 45% in the total cohort, suggesting that these results should remain applicable regardless of histology.

Second, due to its retrospective nature and the structure of the data, it does not allow to assess the cause of working inability, for example, inability to work due to progression or treatment-related toxicities, transition into early retirement, or a personal choice triggered by the generally chronic nature of mIDH glioma.^46^ Although considering the young age of these patients, it is likely that disease progression and oncologic treatments played a central role in the reductions in ability to work through emerging cognitive and communication impairments, fatigue, and increasing seizure burden.

Third, as the DREAM register captures sick leave based on payments by the Danish municipalities, patients with short-term sick leave that is paid by the employer or who are not eligible for sick-leave benefits will not be recorded with sick leave. This could potentially lead to an underestimation of sick leave for these patients. Another limitation of the DREAM register is that it captures weekly sick benefit information, rather than the exact start and end dates of the sick leave, thus limiting the granularity of our results.

Furthermore, data extracted from the research platform were communicated in an aggregated form, and no information or exact patient counts were extracted for groups with less than 5 patients. Consequently, data extracts are subject to both direct and secondary disclosure, leading to some reported lacking precision.

Finally, some uncertainty remains regarding the classification of all study patients as grade 2. The codes used in the Pathology and Cancer Register ensure that all patients have grade II^51^ diffuse astrocytomas (ICD-O-3 9400/3) or oligodendrogliomas (ICD-O-3 9450/3), and a confirmed IDH mutation. Furthermore, since these patients were not directly referred to RT/CT after initial surgery, we expect that they would be classified as grade 2 according to the WHO 2021 classification.^3^

In conclusion, the study demonstrates that grade 2 mIDH glioma patients managed with AO after initial surgery experience significant challenges in workforce participation, with increased sick leaves and productivity losses as the disease progresses, especially after RT/CT initiation. These findings highlight the need for novel treatment strategies to maintain patients’ functionality and quality of life for as long as possible, not only benefiting the patients but also mitigating the societal economic burden.

## Supplementary material

Supplementary material is available online at *Neuro-Oncology Practice* (https://academic.oup.com/nop/).

npaf092_suppl_Supplementary_Tables_1-2

## Data Availability

No patient-level data can be made available to other researchers due to Danish data legislation.
