# Supplementary material for: Evaluating work activity and societal burden in patients with grade 2 IDH-mutant glioma
Source: Neurooncol Pract. 2025 Sep 1;13(2):331–41. doi: 10.1093/nop/npaf092 (PMC13153697; doi:10.1093/nop/npaf092)
Supplement: npaf092_suppl_Supplementary_Tables_1-2 [file npaf092_suppl_supplementary_tables_1-2.docx]

**SUPPLEMENTARY FILES**

Table S1. Overview of data sources and codes used to identify diagnosis, IDM mutation status, brain surgeries, treatments, sickness benefit

| Register name | Content | Years | Variables | Codes used |
| --- | --- | --- | --- | --- |
| National Patient Register | In- and outpatient specialty care | 1 January 2008 – April 2024 | *Diagnosis codes (ICD-10), procedure codes (treatments, imaging, surgeries etc), dates and types of visit and costs* | ***Codes used for identification of brain surgery****:*  *KAAA10* (Biopsy through craniotomy), KAAB00* (Extirpation of intracranial lesion), KAAB10* (Partial excision of intracranial lesion), KAAG00* (Stereotactic intracranial lesion), KAAA99* (Other diagnostic intracranial operation), KAAJ20* (Excision of epileptic focus), KAAE45* (Subtemporal excision or resection of intracranial pathological tissue)*  ***Codes used for identification of treatments (Procedure code)***  *BWGC* (Radiation therapy), BWHA* (Chemotherapy), BXB* (Palliative)* |
| Registry of Medicinal Product Statistics | Dispensed prescription medications | 1 January 2008 – June 2023 | *Prescription and dispensing dates, type of medication (ATC codes), strengths and dosage info* |  |
| National Cancer Register | Cancer cases at diagnosis | 1 January 2008 – 31 December 2022 | *Dates of diagnosis, diagnosis (ICD-10 and ICD-O-3) and cancer stage (TNM)* | ***Codes used for diagnosis:***  *9450/3 (Oligodendroglioma) ; 9400/3 (Diffuse astrocytoma)* |
| National Hospital Medication Register | Hospital-administered drugs | 1 June 2018 – May 2024 | *Treatments administered at hospitals, type of medication (ATC codes), strengths and dosage info* | ***Codes used for identification of chemotherapies (ATC code)***  *L01* (Antineoplastic agents)* |
| Pathology Register | Pathology results from biopsies/tissue | 1 January 2008 – May 2024 | *Results from biopsies including morphology, anatomical localization, grading, molecular test results – all SNOMED based - and dates of biopsy* | ***Codes used to identify mIDH***  *M94503 (Oligodendroglioma IDH mutation and 1p19q co-deletion), M94003*  *(Astrocytoma IDH mutation), F29881(IDH1 positive), FE15R2 (IDH1 gene insertion), FE15R3 (IDH1 gene mutated), FE15R4 (IDH1 gene deletion), FE15R5 (IDH1 gene amplification), FE15S2 (IDH2 gene insertion), FE15S3 (IDH2 gene mutated), FE15S4 (IDH2 gene deletion), FE15S5 (IDH2 gene amplification), M632D0 (1p19q co-deletion), M633A1 (1p normal), M633A2 (1p deletion), M633S1(19q normal), M633S2 (19q deletion)* |
| Laboratory Register | Laboratory results | 1 January 2015 – May 2024 | *Lab data such as lipids, blood glucose and renal tests* |  |
| DREAM | Labour market attachment | January 2008 – March 2024 | *Work inactivity data and type of benefits received on a weekly basis* | ***Benefit codes to identify whether a patient has received sickness benefit or disability pension in a given week***  *890, 893-899, 774, 783*  ***Codes to capture if the patient has left or leaves the labour market due to retirement***  *785-786, 998*  ***Codes to capture if the patient is a student***  *651, 652* |
| Population Register | Demographics | 1 January 2015 – April 2024 | *Date of birth, sex, marital status, region of residence, emigration, immigration, death, origin of country* |  |

Table S2. Definition of time periods

| Time period in general | Operational definition |
| --- | --- |
| Baseline period (9–21 months) | The time period before index date in which background data was collected, e.g., comorbidity, prior diagnosis history etc. |
| Index date | The date of the first pathological requisition describing morphology and/or IDH mutation status of the glioma |
| Follow-up period | Time period after index date in which outcomes data were collected.  From index date until censoring event |
| Censoring event | Patients removed from the study at the event of death, emigration, or end of study period |
| Identification period | The period where patients were included.  1 January 2010 to 31 March 2022 |
| Study period | The overall period during which patients were studied, and data was used to characterize them.  January 2008 to March 2024 |
| Time periods for study outcomes | |
| Baseline period (9–21 months) | The time period before index date in which background data was collected, e.g., comorbidity, prior diagnosis history etc. |
| Post surgery | 3 months post-surgery. Set from Index date and any subsequent surgery date to 3 months after. Occurs nested within the below described periods. |
| Active Observation | The time period from the index date until 1^st^ treatment start or censoring event, excluding any post-surgery period (Initial surgery and re-surgery including their post-surgery periods are nested within this period without it being considered a new treatment). This period was at least 6 months as a result of the exclusion of patients in immediate need of RT/CT or PC. |
| 1^st^ RT/CT (1^st^ Treatment  RT/CT or palliative care) | A new treatment was defined as a record of one of the following after AO period: Chemotherapy; or Radiation or Palliative treatment.  Set from 1^st^ treatment record to end of 1^st^ treatment (A treatment gap of at least 12 weeks) or censoring event. |
| Monitoring after 1^st^ RT/CT | The monitoring period from end of “1^st^ Treatment” to the beginning of “≥2^nd^ Treatment”.  Surgery during this period launch a nested 3-month post-surgery period where outcomes are recorded separately from non-post-surgery periods during monitoring. |
| ≥ 2^nd^ RT/CT (≥ 2^nd^  RT/CT or palliative care | Set from intervention start to end of intervention or censoring event. New treatment is defined as a record of one of the following, after a minimum of 12 weeks gap since the previous treatment: Chemotherapy or Radiation or Palliative treatment. |
| Monitoring after 2^nd^ RT/CT | Set from the end of the 2^nd^ treatment to censoring event.  Surgery during this period launch a nested 3-month post-surgery period where outcomes are recorded separately from non-post-surgery periods during monitoring. |
